# Oral Findings of Rothmund-Thomson Syndrome

**DOI:** 10.1155/2013/935716

**Published:** 2013-11-30

**Authors:** Emin Murat Canger, Peruze Çelenk, İnci Devrim, Aysun Avşar

**Affiliations:** ^1^Department of Oral and Maxillofacial Radiology, Faculty of Dentistry, Erciyes University, Melikgazi, 38039 Kayseri, Turkey; ^2^Department of Oral and Maxillofacial Radiology, Faculty of Dentistry, Ondokuz Mayis University, 55139 Samsun, Turkey; ^3^Department of Periodontology, Faculty of Dentistry, Ondokuz Mayis University, 55139 Samsun, Turkey; ^4^Department of Pediatric Dentistry, Faculty of Dentistry, Ondokuz Mayis University, 55139 Samsun, Turkey

## Abstract

Rothmund-Thomson syndrome (RTS) is an extremely rare genetic condition exhibiting some dermatological, craniofacial, ophthalmological, and central nervous system abnormalities. It has an autosomal, recessive inheritance and its signs begin at childhood. Essential dermatological alteration is poikilodermatosis. A large head with an frontal bossing and broad low nasal bridge has been described in patients with RTS. Bilateral juvenile cataract is a characteristic finding of patients with RTS. Most of the patients have been markedly short and the growth retardation has been proportionate. Mental retardation is a rare condition. An 11-year-old girl who had been previously diagnosed with RTS was consulted with a chief complaint of delaying in tooth eruption. Intraoral examination revealed median rhomboid glossitis in addition to hyperkeratotic tongue. This report aimed to not only present intraoral findings of RTS, but also to demonstrate the lingual findings of a patient with RTS.

## 1. Introduction

Rothmund-Thomson syndrome (RTS) is an autosomal recessive dermatosis which comes into existence in infancy with a characteristic facial rush (poikilodermatous) [1–4]. RTS was first introduced by Rothmund in 1863. The form of RTS with a genetic trait, showing hypogonadism and without cataracts, was introduced in 1923 by Thomson as “Poikiloderma congenitale.” Those two similar syndromes were first mentioned as Rothmund-Thomson by Taylor in 1957 [1–4].

The diagnostic hallmark and heterogeneous clinical features include short stature, sparse scalp hair, sparse or absent eyelashes and/or eyebrows, juvenile cataracts, skeletal abnormalities, radial ray defects, and premature aging. RTS is extremely rare; although its exact prevalence is unknown, to date about 300 cases have been reported [1–4]. It is also suggested that RTS has a genetic trait [1, 4].

The first signs of RTS are of dermatological origin. The main dermatological alteration is poikilodermatosis, which is patch-like pigmentation which usually develops between the age of 3 and 6 months as erythema, with swelling and blistering on the face [1–4].

Ocular signs are accepted as minor signs specific to the Rothmund-type subset of RTS. Juvenile cataracts appearing bilaterally are a characteristic finding of the patient with RTS. They start rapidly and vision disappears within weeks. Other ocular findings are congenital glaucoma, corneal atrophy, colobomah atrophy in the iris and retina, microphthalmia, and photophobia [1–6].

Hypogonadism is evident in about 25% of patients with RTS. Additionally, the majority of female patients present with a menstrual disorder [1].

This case presented alterations in the tongue as well as the known intraoral findings typical of RTS.

## 2. Case Report

An 11-year-old girl attended our department with the chief complaint of delayed eruption of her teeth. From her history, it was learned that deciduous teeth extraction had been performed, and no artificial replacement had occurred.

Her medical history revealed that she was born prematurely. The patient's family members were normal; no consanguinity or blood incompatibility was reported. In addition, her sister was normal. She had a developmental retardation and was 1-2 years behind her chronological age. Her pediatricians had diagnosed RTS when the patient was 2 years old. At the date of attendance, she was suffering from splenomegaly and lacrimal obliteration.

**Figure 1:**
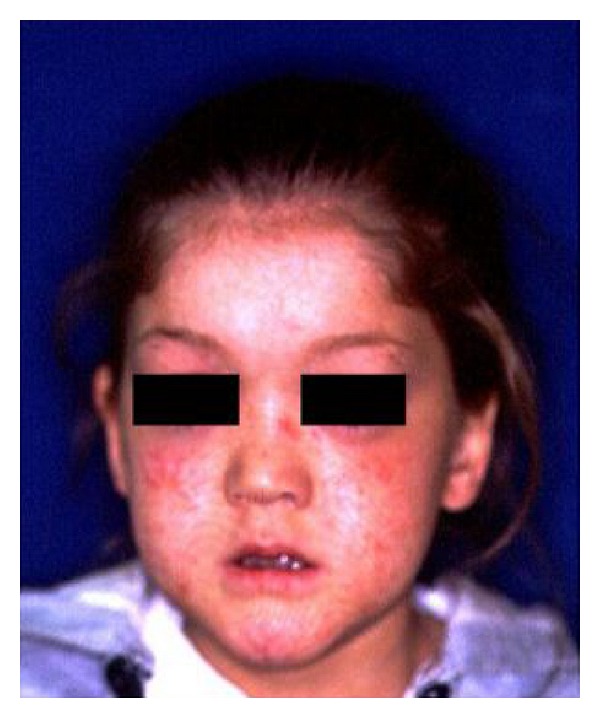
Telangiectatic appearance of the face of patient.

**Figure 2:**
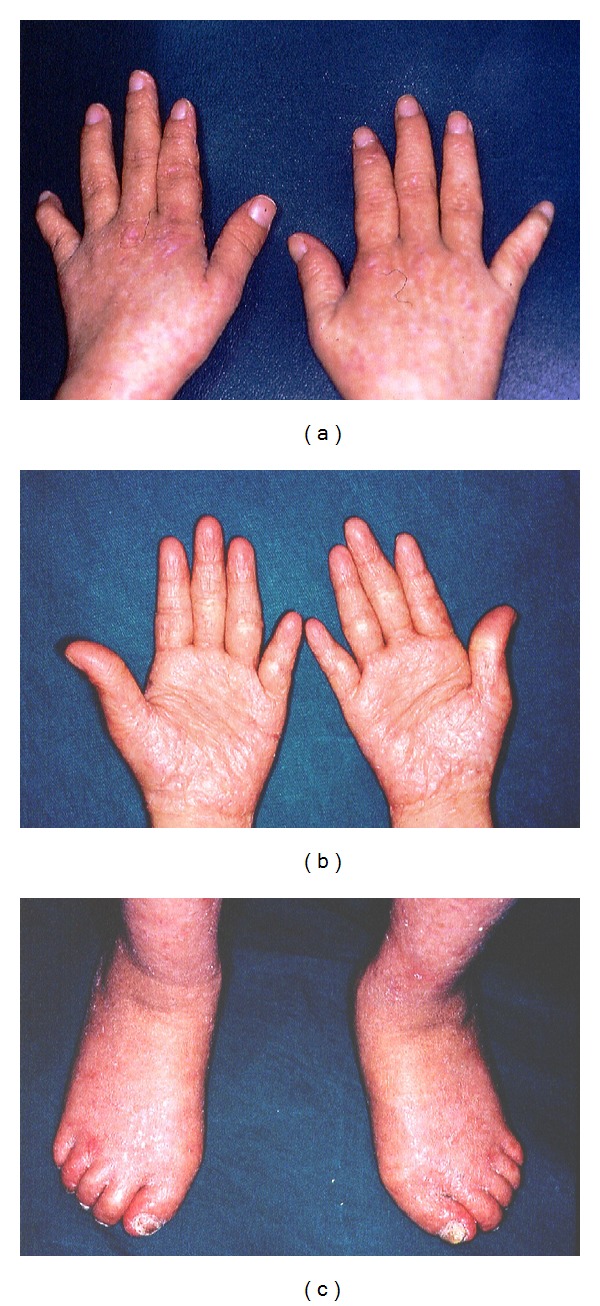
(a) The appearance of the outer surface of patient's hand. (b) The hyperkeratotic appearance of the palm. (c) The hyperkeratotic appearance of the foot.

Our patient had pronounced difficulty in walking and speaking. Extraoral examination revealed exophthalmia and xerophthalmia. Telangiectasia of the face and compressed nose were found (Figure 1). Dermatological findings included macular lesions on the forearm and the front part of the chest, as well as hyperkeratotic depigmented areas on the palms and foot (Figures 2(a), 2(b), and 2(c)).

**Figure 3:**
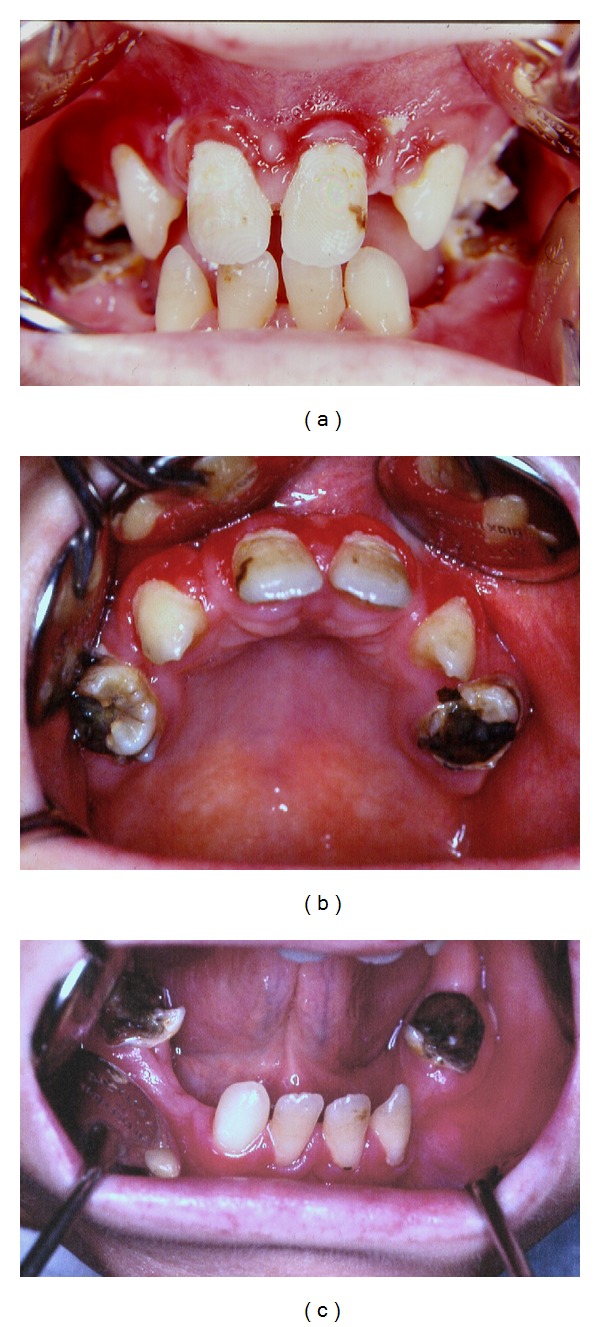
(a) The appearance of the edematous gingival and hyperplasic papillae. (b) The appearance of the maxilla reveals that only right and left first molars, canine, and central incisor teeth are present. Also widespread caries are seen on first molars. (c) The appearance of the mandible reveals that only right and left first molars, lateral incisor, and central incisor teeth are present. Also widespread caries are seen on first molars.

Intraoral examination disclosed hyperemic and edematous gingiva with hyperplasic papilla. There was evident oligodontia; only the teeth charted below were present in the patient's mouth (Figure 3(a)):

$\;\;\;\;\;\;\;\;\;\;\;\;\;\;\;\;\begin{array}{cccccc} 6 & 3 & 1 & 1 & 3 & 6\\ 6 & 2 & 1 & 1 & 2 & 6 \end{array}$

There were widespread carious lesions in all present teeth and coronal destruction due to caries in all permanent first molars (Figures 3(b) and 3(c)).

**Figure 4:**
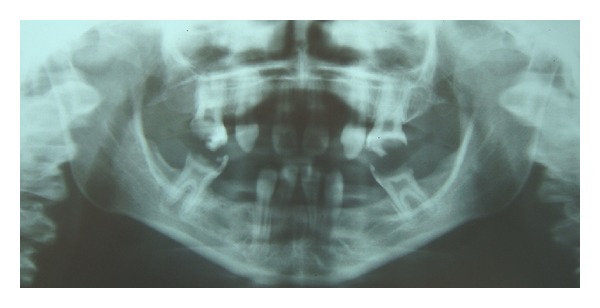
Oligodontia is clearly observed on panoramic radiograph.

The following radiograms were obtained: panoramic full mouth and periapical, lateral, cephalometric hand and wrist. Radiographic examination of the hand and wrist radiograms revealed that the bone age of the patient was consistent with the age of 8. The panoramic and full mouth periapical radiograms revealed neither impacted teeth nor germs of teeth. The alveolar bone, periodontal membranes, and lamina dura of the present teeth were radiographically normal (Figure 4).

**Figure 5:**
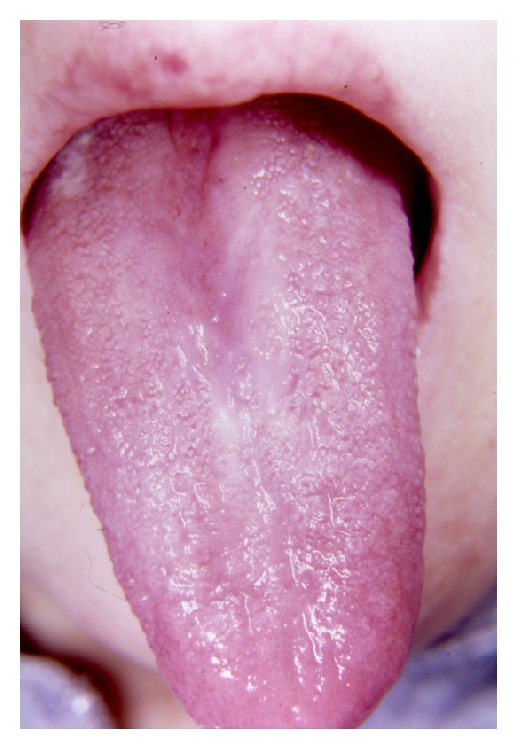
Median rhomboid glossitis on the dorsum of tongue.

In addition to the findings described above, median rhomboid glossitis and hyperkeratosis were evident on the patient's tongue (Figure 5).

The patient was originally referred to the periodontology department seeking therapy for gingivitis and later to pediatric dentistry for restorative procedures and construction of a space retainer. Periodontal and restorative treatments were completed and oral hygiene controls are in progress.

## 3. Discussion

In the Rothmund type of RTS, there are juvenile cataracts; in the Thomson type of RTS, this finding is absent [1]. In our case, although lacrimal obliteration was present as an ophthalmological finding, the cataracts were absent, so the patient can be accepted as a member of the Thomson type of RTS.

It is unclear whether RTS has a sex predilection. Although the patient in our case was female and some research has suggested that RTS is more prevalent in women, other researches found male predominance, so sex predominance has not yet been proven. Further, although certain gene mutations may exist within defined populations, no ethnic predominance has yet been described [1, 2].

Growth delay and the resulting short stature are among the major clinical signs of RTS. Patients are proportionally small; hands and feet are also smaller than normal. Furthermore, bilateral absence of metacarpal and phalanx in the thumbs, deformity of the radius and ulna, and agenesis of the radius are present. Kyphoscoliosis has also been reported. Additionally, osteoporosis, cystic areas resembling fibrous dysplasia, and osteogenesis imperfecta may also be seen [1, 6]. Our patient was proportionally small.

In approximately 50% of RTS cases, hair, eyebrows, and eyelashes are scarce [1–6]. Patients with RTS have a large head with a frontal bossing and a saddle nose. Microcephaly and a triangular face may also be seen [1, 2, 4].

Mental retardation is a rare condition which is seen in approximately 10% of RTS patients. According to some researchers, cases with advanced growth retardation, skeletal anomalies, and ectodermal dysplasia, but without cataracts, constitute a subgroup of the syndrome, and mental retardation is seen prevalently in this subgroup [1, 4–6]. Since cooperation was easily obtained from our patient and her education level was consistent with her coequals, mental retardation was not thought to be present in this case.

Anomalies of teeth were first described by Rothmund in his original article. Overall incidence of dental anomalies has been estimated at between 27% and 59% of cases. Intraoral findings of RTS are listed as microdontia, rudimentary, or hypoplastic teeth, multiple crown malformations, short and conical teeth, increase in prevalence of caries, malocclusion, hypodontia/oligodontia or hyperdontia, ectopic eruption, and delay in eruption. Bifid uvula also has been reported [1, 3, 5, 7]. Our case presented oligodontia. The crowns and roots of teeth were normal. Additionally, widespread and advanced carious lesions were present on both permanent first molars.

Our patient's gingiva was hyperemic and edematous, with hyperplasic papilla. In studies of the periodontal tissue destruction in RTS, researchers suggested that the destruction may be related to apoptosis disorders. Apoptosis is a process which removes dysfunctional and undesirable cells from tissues and differs from necrosis. Apoptosis provides a balance between cell proliferation and cell death to achieve growth and proliferation of normal tissues, while exiguous or extreme apoptosis results in death [3].

Cutaneous and noncutaneous malignancies are among the complications of RTS. Osteosarcoma is the most common noncutaneous malignancy, followed by fibrosarcoma, parathyroid adenoma, Hodgkin's sarcoma, and gastric sarcoma. Cutaneous squamous-cell carcinomas have also been reported, as well as noncutaneous squamous-cell carcinoma of the tongue [8].

Median rhomboid glossitis (central papillary atrophy) is a form of erythematous candidiasis which is seen as a well-demarcated erythematous zone in midline, posterior, and dorsal region of the tongue. As its etiology, embryological fault in the fusion of the two lateral processes of the embryological tongue, and covering the central structure from the first and second branchial arches, the tuberculum impar. Generally it is asymptomatic and needs no treatment, but if there is discomfort or irritation, antifungal agents will be administered [9, 10]. Any cases of median rhomboid glossitis in conjunction with RTS couldnot be found. There is only one case of squamous-cell carcinoma of the tongue in a patient of RTS which was written by Marín-Bertolín et al. [8]. Differential diagnosis of RTS is very difficult due to the presence of some identical signs in other syndromes (e.g., Werner, Cockayne, Bloom, and Kindler-Gottner (*Acrogenia*)) and some other poikilodermatosis (e.g., geoderma dysplastica, hereditary sclerosing poikiloderma, dyskeratosis congenita, and xeroderma pigmentosum) [1, 2].

## References

[1] Gorlin RJ, Cohen MM, Hennekam RCM (2001). *Syndromes of the Head and Neck*.

[2] Larizza L, Roversi G, Volpi L (2010). Rothmund-thomson syndrome. *Orphanet Journal of Rare Diseases*.

[3] Haytaç MC, Öztunç H, Mete UÖ, Kaya M (2002). Rothmund-Thomson syndrome: a case report. *Oral Surgery, Oral Medicine, Oral Pathology, Oral Radiology, and Endodontics*.

[4] Pianigiani E, De Aloe G, Andreassi A, Rubegni P, Fimiani M (2001). Rothmund-Thomson syndrome (Thomson-type) and myelodysplasia. *Pediatric Dermatology*.

[5] Güler O, Aydın M, Uğraş S, Kışlı E, Metin A (1998). Rothmund-Thomson syndrome associated with esophageal stenosis: a case report. *Surgery Today*.

[6] Kerr B, Ashcroft GS, Scott D, Horan MA, Ferguson MWJ, Donnai D (1996). Rothmund-Thomson syndrome: two case reports show heterogeneous cutaneous abnormalities, an association with genetically programmed ageing changes, and increased chromosomal radiosensitivity. *Journal of Medical Genetics*.

[7] Daley TD, Wysocki GP, Bohay RN (1996). Osteopathia striata, short stature, cataracts, and microdontia: a new syndrome? a case report. *Oral Surgery, Oral Medicine, Oral Pathology, Oral Radiology, and Endodontics*.

[8] Marín-Bertolín S, Amorrortu-Velayos J, Aliaga Boniche A (1998). Squamous cell carcinoma of the tongue in a patient with Rothmund-Thomson syndrome. *British Journal of Plastic Surgery*.

[9] Neville BD, Damm DD, Allen CM, Bouquot JE (2002). *Oral and Maxillofacial Pathology*.

[10] Yarom N, Cantony U, Gorsky M (2004). Prevalence of fissured tongue, geographic tongue and median rhomboid glossitis among Israeli adults of different ethnic origins. *Dermatology*.

